# An immunogenetic view of COVID-19

**DOI:** 10.1590/1678-4685-GMB-2021-0036

**Published:** 2021-08-25

**Authors:** Vitor R. C. Aguiar, Danillo G. Augusto, Erick C. Castelli, Jill A. Hollenbach, Diogo Meyer, Kelly Nunes, Maria Luiza Petzl-Erler

**Affiliations:** 1 Universidade de São Paulo, Departamento de Genética e Biologia Evolutiva, São Paulo, SP, Brazil.; 2 University of California, UCSF Weill Institute for Neurosciences, Department of Neurology, San Francisco, CA, USA.; 3 Universidade Federal do Paraná, Departamento de Genética, Curitiba, PR, Brazil.; 4 Universidade Estadual Paulista, Faculdade de Medicina de Botucatu, Departamento de Patologia, Botucatu, SP, Brazil.

**Keywords:** Immunogenetics, COVID-19, SARS-CoV-2, HLA, KIR

## Abstract

Meeting the challenges brought by the COVID-19 pandemic requires an interdisciplinary approach. In this context, integrating knowledge of immune function with an understanding of how genetic variation influences the nature of immunity is a key challenge. Immunogenetics can help explain the heterogeneity of susceptibility and protection to the viral infection and disease progression. Here, we review the knowledge developed so far, discussing fundamental genes for triggering the innate and adaptive immune responses associated with a viral infection, especially with the SARS-CoV-2 mechanisms. We emphasize the role of the HLA and KIR genes, discussing what has been uncovered about their role in COVID-19 and addressing methodological challenges of studying these genes. Finally, we comment on questions that arise when studying admixed populations, highlighting the case of Brazil. We argue that the interplay between immunology and an understanding of genetic associations can provide an important contribution to our knowledge of COVID-19.

## Introduction

The coronavirus disease 19 (COVID-19) caused by the severe acute respiratory syndrome coronavirus 2 (SARS-CoV-2) is imposing severe humanitarian, social, and economic consequences. The ongoing pandemic has affected the lives of millions of people around the world. As of April 2021, Brazil has recorded the third-highest number of COVID-19 cases worldwide, with nearly 15 million infected individuals and the second-highest number of about 400,000 deaths by COVID-19 (WHO COVID-19 | Brazil, 2021).

Two genera of coronaviruses cause human disease: alphacoronaviruses HCoV-229E and HCoV-NL63, and betacoronaviruses HCoV-HKU1, HCoV-OC43, SARS-CoV (presently named SARS-CoV-1), MERS-CoV (Middle East respiratory syndrome), and SARS-CoV-2 (Ogimi et al., 2020). The four HCoV-* viruses cause mild self-limiting respiratory infections, but MERS-CoV, SARS-CoV-1, and the new SARS-CoV-2 may cause significant morbidity and mortality (Song et al., 2019; Ogimi *et al.,* 2020). The most likely natural reservoir of these three viruses are bats, and the possible intermediate hosts are the palm civet for SARS-CoV-1 and the dromedary camel for MERS-CoV (Song *et al.,* 2019; Ogimi *et al.,* 2020). Whether SARS-CoV-2 was transmitted directly from bats to humans or through an intermediate host is still an open question (Lam et al., 2020).

The SARS-CoV-2 is phylogenetically close to SARS-CoV-1, which emerged in 2002 in China and caused more than 8,000 cases in 29 countries over eight months, with a case mortality rate of around 10% (Song et al., 2019). The total number of cases reported for MERS was 2,254 from 2012 through January 2020, with 35% mortality (Song *et al.,* 2019; Rabaan et al., 2020). Comparing data from different diseases and sources is tricky, yet the case fatality rate of COVID-19 is definitely much lower, estimated at 2.2% worldwide (WHO COVID-19 Dashboard, April 13 2021). However, the socioeconomic impact of this disease widely surpasses SARS and MERS, because of the high infectivity and rapid spread of the SARS-CoV-2, and the extreme burden placed on healthcare systems due to the need for hospitalization and artificial ventilation for severe cases. 

**Figure 1 -:**
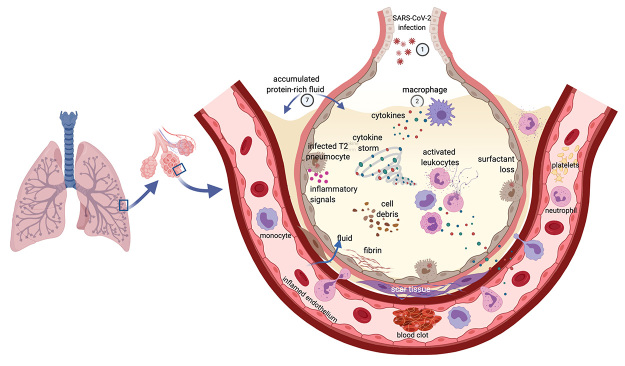
Features of severe COVID-19 and acute respiratory distress syndrome (ARDS) in the lung. SARS-CoV-2 enters the body by the airways and infects lung cells **(1)**. Immune cells, including macrophages **(2)**, and the infected cells **(3)** react to the virus and produce cytokines, interferons, and additional inflammatory signals **(4)**, which attract other leukocytes. These also produce cytokines **(5)** that may lead to hyper inflammation and the “cytokine storm” **(6)**. The inflamed capillaries allow fluid to sweep into the alveoli and fill the lung cavities **(7)**. Damage to the lung occurs through several processes, including the consequences of surfactant loss **(8)**, the accumulated liquid, and the formation of fibrin and scar tissue **(9)**. With the participation of complement components, coagulation factors, neutrophils, and platelets, blood clots are formed in the inflamed blood vessel **(10)**. The association between thromboembolic events and higher levels of von Willebrand’s factor (vWF) and factor VIII with non-group O (see main text) may underlie the association of blood group A with increased susceptibility to severe COVID-19. The deregulated immune response and disturbed coagulation spark inflammation throughout the body damaging other organs and fuels the respiratory failure responsible for most deaths caused by COVID-19. Moreover, the virus may evade the immune response by blocking the effect of immune cells and soluble as well as membrane-bound mediators of immunity and complete its cycle in infected cells, spreading throughout the body. Figure created with Biorender.

The symptoms after infection by SARS-CoV-2 range from asymptomatic to severe disease and death (McIntosh et al., 2020; Wu and McGoogan, 2020). The most common clinical symptoms are fever, dry cough, dyspnea, fatigue, dysgeusia, and anosmia (taste and smell disorders). Other common symptoms are myalgia, rhinorrhea, sore throat, diarrhea, nausea and/or vomiting, and headache. About 15% of patients develop severe disease with exuberant inflammatory response, lymphopenia, thromboembolic complications, and hypoxemia that eventually leads to acute respiratory distress syndrome (ARDS) and multiple organ dysfunction syndromes (MODS) (McIntosh *et al.,* 2020; Wu and McGoogan, 2020). Patients may also experience arrhythmias, acute cardiac injury, kidney injury, liver dysfunction, or neurologic manifestations. Possible neurological damage after SARS-CoV-2 infection, even among recovered patients, increases its impact on the healthcare system (Ellul et al., 2020; Kotfis et al., 2020; Moriguchi et al., 2020; Varatharaj et al., 2020; Zanin et al., 2020). Some features of severe COVID-19 and ARDS are presented in Figure 1.

The risk factors for severe illness are older age, male sex, and medical comorbidities such as diabetes mellitus, cardiovascular disease, hypertension, chronic kidney disease, cancer, obesity, and smoking (Espinosa et al., 2020; McIntosh et al., 2020; Wu and McGoogan, 2020). However, these factors do not explain all the variation, as exemplified by reports of young individuals without comorbidities, including children, that developed severe forms of the disease (e.g., van der Made et al., 2020) and several anecdotal reports of elderly patients with other illnesses that fully recovered from COVID-19. Undoubtedly, host genetics plays a pivotal role in influencing the human response to infection, and rare as well as polymorphic genetic variants are expected to underlie the consequences of SARS-CoV-2 infection. Understanding the role of immune-related genes in the response to SARS-CoV-2, as well as the distribution of associated genetic variants in populations across the world, is a critical element in understanding the disease and in seeking strategies to respond to it. 

Human populations have been locked in a coevolutionary arms race with pathogens for thousands of years, with natural selection favoring protective genetic variants against pathogenic viruses, bacteria, and other microorganisms. The findings that as many as 30% of all adaptive amino acid changes in the human proteome are related to selective pressures imposed by viruses underscores the critical participation of viruses in this process (Enard et al., 2016).

Pathogens also evolve in response to host adaptive changes, increasing their capacity to evade the immune response, and to invade, multiply, and be transmitted to other hosts. As a result, the host-pathogen arms race is a dynamic process that leaves signatures in the human genome (reviewed in Karlsson et al., 2014). Therefore, identifying these traces allows discovering genes involved in adaptive response against pathogens (Fan et al., 2016).

Scans for natural selection signatures have identified a conspicuous enrichment of genes related to the immune function in the human genome (Hedrick, 2002; Sabeti et al., 2006; Andrés et al., 2009; DeGiorgio et al., 2014; Bitarello et al., 2018). The group of immune-related genes identified to be under selection is broad, encompassing components of both adaptive and innate immune responses (Akey et al., 2004; Fumagalli et al., 2011). These results show that immune responses against pathogens have been critical for local adaptation of human populations since ancient times. While scans for selection only identify genomic signatures shaped by selection in the past, it is much more challenging to identify evidence of ongoing natural selection.

Many studies have surveyed the association between host genetic variation and susceptibility to infection or disease outcomes. The associations between genetic variants and response to pathogens are the contemporary counterpart of the selective process that generates the evolutionary signatures. Not surprisingly, genes related to immune responses systematically appear in association studies of infectious diseases, with specific variants associated with increased susceptibility, while others are associated with the protection from different diseases (Hill, 2012). 

In this review, we discuss the importance of association studies focusing on variation in genes related to immune function, emphasizing HLA (*human leukocyte antigen*) and KIR (*killer-cell immunoglobulin-like receptor)* gene families when searching for host determinants of the response to SARS-CoV-2, without ignoring the paramount importance of other genes involved in fighting viral infections and diseases. The prominent role of the HLA and KIR genes in the viral immune responses, and of HLA in vaccine development, make them natural candidates in the search for COVID-19 disease-relevant variants. We draw attention to the peculiar characteristics of these gene families (e.g., their high polymorphism, distinct allele frequencies in worldwide populations, and their interaction with each other), which impose technical challenges especially for genomewide studies and therefore demand targeted strategies. We list and discuss these challenges and alternative approaches to minimize errors in association studies. Finally, we introduce some research strategies and how the scientific community has been engaged to combine resources, share data, and accelerate the knowledge about the immunogenetics of COVID-19. 

## HLA in viral infections

The HLA molecules were initially investigated due to their determinant role in allogeneic transplantation outcomes (Dausset, 1958; Klein, 1986; Thorsby, 2009), but their major functions are immunomodulation and the triggering of adaptive immune responses (Bjorkman et al., 1987; Brown et al., 1993; Jones et al., 2006). The classical HLA class I molecules (class Ia), HLA-A, HLA-B, and HLA-C, are expressed in all nucleated cells and present peptides of cytosolic origin to CD8^+^ T lymphocytes, and together with accessory signals stimulate a cytotoxic response against target cells. The non-classical HLA class I molecules (class Ib) are expressed in specific tissues and their primary function is immunomodulation (Donadi et al., 2011; Kraemer et al., 2014; Persson et al., 2020). In contrast, the classical HLA class II molecules (HLA-DR, HLA-DQ, and HLA-DP) are expressed by antigen-presenting cells and present exogenous antigens to CD4^+^ T lymphocytes, which in the context of costimulatory signals trigger adaptive immune responses. 

The HLA genes are located within the human major histocompatibility complex (MHC), in chromosome region 6p21.3 (Klein and Sato, 2000), and are extraordinarily polymorphic (Robinson et al., 2020) with thousands of alleles in some loci (IPD-IMGT/HLA | Statistics, 2020). The HLA genotypes directly influence the range of antigens presented by a given individual to the T lymphocytes and how the HLA molecules interact with other receptors on NK cells. Consequently, there is variation among individuals regarding the set of viral peptides that they can present and eventually create an efficient response to.

Infectious diseases are one of the leading causes of human mortality (Burgner et al., 2006) and a major selective pressure for human survival (Sabeti et al., 2002; Frodsham and Hill, 2004; Walsh et al., 2006). Among the plethora of genes involved in human immune responses, HLA variants are among those with the strongest reported associations with infection risk and progression (Cooke and Hill, 2001; Tian et al., 2016). 

A well-documented example of how HLA alleles influence viral infections is HIV (*human immunodeficiency virus*) infection outcome (Fellay et al., 2007; Kawashima et al., 2009; International HIV Controllers Study *et al.,* 2010). While some HLA-B molecules can accommodate specific HIV antigens and trigger an immune response, others cannot. The homozygosity of class Ia genes was also strongly associated with rapid progression of HIV infection (Carrington et al., 1999; Tang et al., 1999) while differential HLA expression levels were associated with HIV viral load control (Apps et al., 2013; Kulkarni et al., 2013; Ramsuran et al., 2018). Moreover, the HLA variation has been also associated with hepatitis B, hepatitis C, and several other infectious diseases (Blackwell et al., 2009). 

During the SARS-CoV-1 (2002-2003) and MERS-CoV (2012) epidemics, several studies demonstrated that HLA alleles were associated with differential susceptibility, but these results were not consistent among studies (Sanchez-Mazas, 2020). The discrepancies are probably explained by the fact that SARS-CoV-1 and MERS-CoV transmission was in specific geographic regions (Asia and the Middle East) and the consequently limited pool of HLA alleles identified in those populations. Due to the pandemic nature of SARS-CoV-2, case-control studies are required to assess a broader range of HLA alleles, potentially revealing new sets of associated alleles differing among geographic regions and populations. Different populations may present distinct alleles associated with susceptibility, depending on the pool of HLA alleles present in each population. Besides differences in study design and statistic power issues, this may be a reason why thus far no conclusive associations between COVID-19 and HLA have been reported (Ellinghaus et al., 2020; Novelli et al., 2020; Wang et al., 2020a,c; Amoroso et al., 2021; Anzurez et al., 2021; Leite et al., 2021; Lorente et al., 2021; Shkurnikov et al., 2021; Yung et al., 2021).

### Mapping the potential response to SARS-CoV-2 mediated by HLA peptide presentation

Understanding the repertoire of viral epitopes that specific HLA allotypes can bind provides a mechanistic basis for interpreting genetic associations and contributes to developing vaccines and identifying viral escape epitopes (reviewed in Gfeller and Bassani-Sternberg, 2018).

Despite significant methodological advances in the experimental screening of peptide repertoires presented by HLA (e.g., mass spectrometry and *in vitro* binding assays), only a few HLA allotypes have been studied (Bassani-Sternberg et al., 2015; Caron et al., 2015; Gfeller and Bassani-Sternberg, 2018). Experimental data, together with genetic sequences from pathogens in combination with HLA alleles, make up the reference databases for predictive computational methods (e.g., machine learning, neural network). The success of the computational prediction depends on the availability of large-scale training datasets (Abelin et al., 2017; Dhanda et al., 2019) and the accuracy of the prediction models (Rivino et al., 2013). 

SARS-CoV-1 and MERS-CoV experimentally-determined epitopes can be found in several public databases (e.g., the Virus Pathogen Database and Analysis Resource, ViPR (Pickett et al., 2012); The Immune Epitope Database, IEDB (Vita et al., 2019)). Due to their genetic similarity, several studies have used the information obtained experimentally for SARS-CoV-1 to make predictions for SARS-CoV-2 (Ahmed et al., 2020; Grifoni et al., 2020; Lee and Koohy, 2020). However, Ahmed *et al.* (2020) showed that only 23% of known SARS-CoV-1 and SARS-CoV-2 T-cell putative epitopes are identical. Even though part of the SARS-CoV-2 epitope information may not be captured in these comparisons, regions that are identical in SARS-CoV-1 and SARS-CoV-2 are possibly those with a low substitution rate. Consequently, vaccination strategies designed to target the immune response toward these conserved epitope regions could generate immunity that is cross-protective to SARS-CoV-2 and also to other coronaviruses (Grifoni *et al.,* 2020).

HLA affinity prediction for SARS-CoV-1 has been studied based on a limited number of alleles (e.g., HLA-A*02:01, HLA-A*11:01, HLA-A*24:02), identifying potential affinities with epitopes from spike (S) and nucleocapsid (N) proteins (Tsao et al., 2006; Rivino et al., 2013). A later study applied an immunization prime-boost strategy to increase the number of memory CD8^+^ T-cells in the respiratory tract, finding that structural proteins S and N are highly immunogenic and induce longer-lasting neutralizing antibodies than other coronavirus proteins (Channappanavar et al., 2014). Nowadays, many studies of SARS-CoV-2 focus on antigens from these viral structural proteins (Kiyotani et al., 2020; La Porta and Zapperi, 2020; Sanami et al., 2020), as well as on non-structural proteins (Marchan, 2020).

**Figure 2 -:**
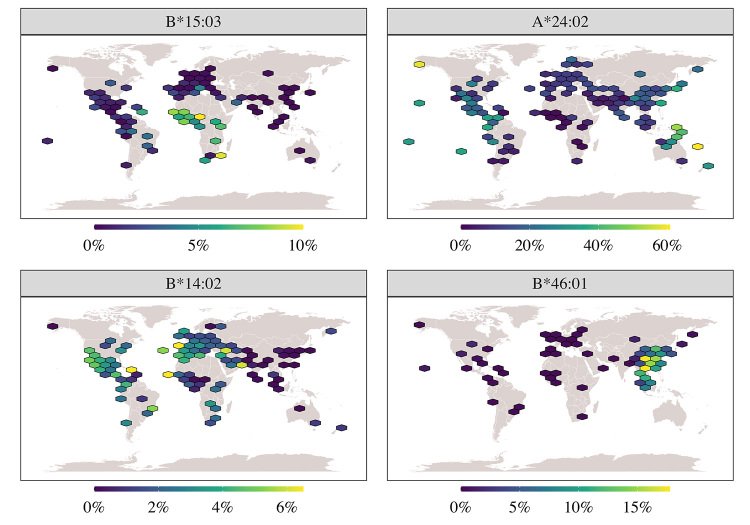
Global distribution of HLA alleles that are either strong or weak binders of SARS-CoV-2 epitopes. **Upper left panel**: The frequency of the predicted SARS-CoV-2 strong binder HLA-B*15:03 is high in African populations (usually > 8%) and rare among Europeans, Asians, and Americans. **Lower left panel**: The frequency of the predicted SARS-CoV-2 weak binder HLA-B*14:02 is high in American populations, particularly Brazil, and also among European and Africans. **Upper right panel**: The frequency of the cosmopolitan allele A*24:02. **Lower right panel**: The frequency of the Asian-restricted B*46:01 allele. Frequency data were obtained from the Brazilian and 1000 Genomes high-coverage sequencing data processed with specific HLA bioinformatics workflow (Naslavsky et al., 2020), and from the allelefrequencies.net website (Gonzalez-Galarza et al., 2020).

At first, many studies of SARS-CoV-2 were limited to presenting a predicted list of potential candidate epitopes with high affinity to certain HLA allotypes (Grifoni et al., 2020; Kiyotani et al., 2020; Lucchese, 2020; Vashi *et al.,* 2020). However, studies have recently taken a step forward and started evaluating other characteristics of HLA+epitope complexes, such as antigenicity, toxicity, and population coverage (Joshi et al., 2020; Mukherjee et al., 2020; Yarmarkovich et al., 2020). HLA-A*68:01, B*15:03, and DRB1*07:01 are recurrent among the lists of HLA allotypes showing a stronger binding with SARS-CoV-2 predicted peptides (Barquera et al., 2020; Joshi *et al.,* 2020; Iturrieta-Zuazo et al., 2020; La Porta and Zapperi, 2020). Conversely, HLA-B*14:02, B*35:03, and B*46:01 have a low predicted binding for SARS-CoV-2 peptides, raising the hypothesis that individuals expressing this molecule may be more vulnerable to COVID-19 (Nguyen et al., 2020). The frequency of each of these HLA alleles varies among populations. For instance, the predicted strong binder HLA-B*15:03 is frequent in most African populations, particularly in Guinea-Bissau and Uganda, and very rare in Europe and Asia (Figure 2, upper right panel). The predicted weak binder HLA-B*14:02 is frequent in Europe, Africa, and America, particularly in Brazil (Figure 2, lower right panel). Other examples include the weak binders HLA-B*35:03, highly frequent in India and Pakistan, and B*46:01, highly frequent and mostly detected in East Asia. This underscores the potential for the genetic basis of response to COVID-19 differing among populations.

Many other HLA alleles were included in some studies but not in others (Barquera et al., 2020; Joshi et al., 2020; Mukherjee et al., 2020; Yarmarkovich et al., 2020; Leite et al., 2021; Shkurnikov et al., 2021). This heterogeneity highlights the methodological differences among studies and the differences in the sets of the selected HLA alleles, which may be restricted to a geographic region in some cases (Kiyotani et al., 2020) (Figure 2, lower left panel), or cosmopolitan (Figure 2, upper left panel) in others (Barquera *et al.,* 2020).

For successful vaccination strategies, it is critical to identify epitopes that can be recognized not only by one but multiple HLA allotypes and, consequently, cover a wide diversity of populations (Ahmed et al., 2020; Barquera et al., 2020). Studies with SARS-CoV-1 drew attention to the binding affinity of viral epitopes based on the functional classification of HLA supertypes (groups of molecules sharing chemical properties in the B and F pockets of the peptide binding region). As expected, allotypes belonging to the same supertype had an affinity to similar viral peptides. In contrast, those belonging to different supertypes had little overlap in the repertoire of viral peptide sets (Sylvester-Hvid et al., 2004). The A3 supertype (HLA-A*03:01 and HLA-A*11:01) has an affinity to the greatest range of SARS-CoV-1 epitopes (Sylvester-Hvid *et al.,* 2004; Blicher et al., 2005).

Certain HLA supertypes are geographically widely distributed. For example, supertype A3 (alleles from the A*03, A*11, A*30, A*31, A*33, A*66, A*68, and A*74 allele groups) is present in at least 44% of the world population (Sette and Sidney, 1999). Moreover, the frequency distribution of supertypes is relatively conserved worldwide (Dos Santos Francisco et al., 2015), which allows the distribution and frequencies of supertypes to also be taken into account in the development of vaccines. In studies for SARS-CoV-2 vaccines, there has been an active effort to identify candidate peptides for which the widely distributed supertypes A3 and B7 have a strong affinity (Kalita et al., 2020), however, these studies are still in the early stages of development.

A major effort of vaccine development is to induce CD8^+^ cytolytic T lymphocytes (CTL) and CD4^+^ T-helper immune responses (Ahlers and Belyakov, 2010), and the HLA+peptide complex plays a crucial role in this process. Vaccine development requires a detailed investigation of how SARS-CoV-2 antigens interact with the immune system. However, experimental approaches require long periods of study, which represents a challenge due to the urgency required for the development of effective COVID-19 vaccines. 

Reverse vaccinology assesses the pathogen genome using bioinformatic tools to predict promising target epitopes. Combining it with HLA binding predictions may be an interesting path for vaccine discovery (Enayatkhani et al., 2020; Ong et al., 2020; Tahir Ul Qamar et al., 2020). However, it selects a reduced set of antigens that can better meet a vaccine’s requirements: activation of the immune response and effectiveness for most individuals in the population. This strategy does not rule out the vaccine development and testing validation steps required by regulatory agencies to prove the safety and effectiveness of vaccines.

## HLA expression in the context of infectious diseases

The differential expression of HLA is also associated with susceptibility to viral infections. For example, higher HLA-C expression leads to a better control of HIV-1 infection (Thomas et al., 2009; Kulkarni et al., 2011; Apps et al., 2013; Bachtel et al., 2018; Parolini et al., 2018); HLA-DP expression has been associated with HBV clearance (Thomas *et al.,* 2012; Ou et al., 2019); HLA-DR levels were shown to correlate with susceptibility to infection by bat Influenza A viruses in human cell lines (Karakus et al., 2019). In a transcriptome-wide association study, Kachuri et al. (2020) identified a predominance of associations in HLA class II genes between expression levels and antibody response to multiple prevalent viruses (such as EBV, Herpes, and polyomavirus 2).

Understanding how HLA expression varies among individuals, alleles, and tissues can illuminate the role of HLA genes in SARS-CoV-2 infection. Many studies have profiled HLA alleles with respect to their ability to present SARS-CoV-2 peptides and have identified strong and weak binders. The integration of such data with expression levels could lead to the identification of HLA alleles which are both strong binders and have sufficient expression levels to efficiently elicit an immune response to the virus. 

The study of HLA expression is an area of active research and can be undertaken using a wide array of methods. Developments include qPCR-based (Ramsuran et al., 2015) and antibody-based (Apps et al., 2013) approaches to estimate mRNA and surface protein levels, respectively, as well as next generation sequencing (NGS) assays (RNA-seq) that aim to estimate HLA expression at the levels of isoforms or HLA alleles (Cole et al., 2020), and bioinformatics pipelines to extract accurate HLA information from standard RNA-seq data for whole transcriptomes (Boegel et al., 2012; Lee et al., 2018; Aguiar et al., 2019; Orenbuch et al., 2020).

However, there is still scarce knowledge on HLA expression in COVID-19. Previous studies of SARS-CoV-1 and MERS-CoV indicate that coronaviruses induce transcription changes in infected tissues, including the modulation of HLA genes (Josset et al., 2013; Menachery et al., 2018). The few studies so far on HLA expression in SARS-CoV-2 infection suggest a down-regulation of HLA expression at the mRNA level (Vastrad et al., 2020; Wilk et al., 2020) and at the protein level (Zhang et al., 2020c). These findings indicate that obtaining expression estimates at different levels (mRNA, protein) may help understand HLA regulation in COVID-19.

Whether and how HLA expression affects SARS-CoV-2 infection will also require the disentanglement of different effects to pinpoint which are causal. For example, the protective effects of some HLA alleles may result from the overall gene expression levels which they mark (Thomas et al., 2009; Thomas *et al.,* 2012; Apps et al., 2013; Wissemann et al., 2013); some HLA GWAS SNPs may be non-independent of HLA eQTLs, and HLA eQTLs may be linked to specific HLA lineages (Aguiar et al., 2019).

It may also be necessary to investigate factors located elsewhere in the genome. For example, HLA-C surface levels were associated with a 3’-UTR microRNA binding site, and variation in the microRNA expression itself influences HLA-C levels (Kaur et al., 2017). For HLA class II genes, CIITA is an important transactivator that affects HLA expression regardless of the HLA allele (Carey et al., 2019). Although CIITA plays additional roles in antiviral responses which are not mediated by HLA (Forlani et al., 2016; Bruchez et al., 2020), previous studies reported a downregulation of both CIITA and HLA in MERS-CoV (Josset et al., 2013; Menachery et al., 2018), thus indicating a putative role of the CIITA-HLA interaction in coronavirus diseases.

## Killer-Cell Immunoglobulin-Like Receptor (KIR) Molecules Bind HLA and Are Essential for Innate Immunity

**Figure 3 -:**
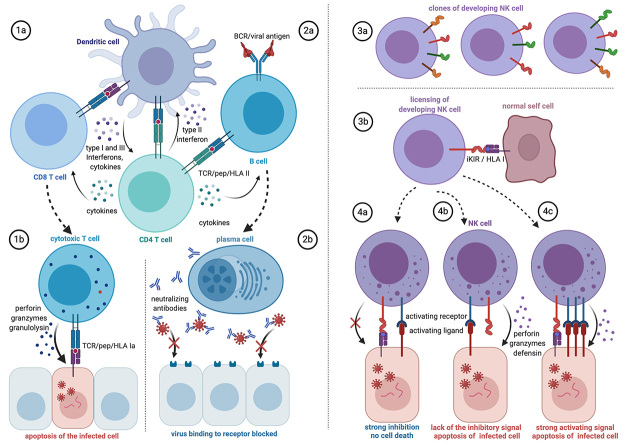
Predicted HLA involvement in T cell, B cell, and NK cell responses to SARS-CoV-2 infection. **Left**: T and B cells are central to adaptive immunity, whose effectivity is influenced by the individual’s HLA genotype. **(1a)** Dendritic cells (DC) present viral peptides bound to HLA Ia and HLA II to, respectively, CD8+ and CD4+ T cell clones displaying specific TCR. (**1b**) The effector cytotoxic CD8+ T cell recognizes the infected target cell by interaction of its specific receptor (TCR) with HLA Ia/peptide on the target and lyses the infected cell. **(2a)** B cells bind viral antigens through specific membrane immunoglobulin receptors (BCR). The internalized antigens go through the class II pathway and HLA II plus viral peptide displayed at the B cell membrane are recognized by the previously primed helper CD4+ T cell to activate the B cells. **(2b)** The activated B cells differentiate in antibody-secreting plasma cells. The neutralizing specific antibodies bind to the virus’s antigen, blocking the viral entry into the cell. Other types of antibodies (not shown) may bind to viral antigens at the surface of infected cells to recruit NK cells or trigger the complement cascade. Besides, all the cell-cell interactions indicated in this schematic view depend on signals by accessory membrane molecules (not shown) and soluble factors such as interferons and specific sets of cytokines. **Right**: The natural killer cells (NKc) are important players in innate immunity. NKc repertoires differ among individuals due to the high polymorphism of*KIR*and*HLA*class I, resulting in differential susceptibility to infection and disease. **(3a)** Each individual has numerous NK cell clones that differ for the number and types of inhibitory and activating KIR receptors. **(3b)** During NKc development, the high-affinity binding of inhibitory KIRs (iKIR) with HLA class I (HLA I) enhances the functions of NKc through a process known as licensing. The strength of the interactions depends on the individual’s *HLA* and *KIR* genotype and dictates the efficiency of mature NK effector function. **(4a)** The KIR/HLA I interaction inhibits apoptosis of the infected cells even in the presence of activating interactions, especially of NKc licensed by strong interactions; **(4b)** signaling by the activating receptor/ligand leads to apoptosis of the infected target cell when HLA I is absent; **(4c)** strong activating signals may overcome the iKIR/HLA I interaction especially if this interaction is weak, resulting in apoptosis of the target. Moreover, in severe COVID-19, NKc often are reduced in number and dysregulated. Figure created with Biorender.

Although the primary focus of HLA research is related to antigen presentation to T cells, certain HLA molecules evolved and specialized as ligands for natural killer (NK) cell receptors, activation of the immune response or modulation of its effectiveness (Kiessling et al., 1975; Herberman and Ortaldo, 1981) (Figure 3). Their cytotoxicity against target cells is mediated by surface receptors that recognize abnormal patterns that are characteristics of infected and neoplastic cells (Parham, 2004; Bottino et al., 2005).

Among the variety of NK cell receptors, the killer-cell immunoglobulin-like receptor (KIR) family stands out as the most polymorphic and most explored in the context of diseases. KIR molecules control NK cells’ activating and inhibitory signals toward target cells and are regulated by interactions with HLA class I molecules. Infection by several pathogens or neoplastic transformation frequently results in abnormal expression of HLA class I on the cell surface. Abnormal HLA expression ultimately affects the balance of activating and inhibitory signals transduced by NK cell receptors, which triggers the cytotoxic response (Ljunggren and Kärre, 1990; Yawata et al., 2008).

The KIR complex is located at the chromosome region 19q13.42 (Wilson et al., 1997; Wende et al., 1999) and consists of 13 genes and two pseudogenes that exhibit an uncommon structural variation of presence and absence of genes and a high allelic polymorphism. There is growing evidence that KIR and HLA are coevolving as a unique and complex system and are important for human survival (Augusto and Petzl-Erler, 2015). Among all HLA class Ia molecules, HLA-C originated more recently and evolved to primarily bind KIR and regulate NK cell response, while HLA-A and HLA-B retained their primary function as T cell ligands (Older Aguilar et al., 2010). Combinations of KIR-HLA have been associated with diseases (Williams et al., 2005; Kulkarni et al., 2008; Augusto, 2016; Boudreau and Hsu, 2018), including infection (Martin et al., 2007; Podhorzer et al., 2017; Alves et al., 2019; Auer et al., 2020), cancer (Middleton et al., 2007; Al Omar et al., 2010; Kim et al., 2014), and autoimmunity (Suzuki et al., 2004; Augusto *et al.,* 2012; Hollenbach et al., 2016; Anderson et al., 2020).

Variation in genes encoding receptors for natural killer (NK) cells deserves special attention in host genetics affecting the susceptibility to any viral infection, including SARS-CoV-2. For SARS patients, low CD3^+^, CD4^+^, CD8^+^, and NK cell counts might be prognostic indicators to predict admission to intensive care unit (ICU) for SARS patients (Chan et al., 2004). The number of NK cells increased with recovery from SARS, but did not return to normal levels even at the 5th week after the disease onset (Dong et al., 2004). Not only were NK cell counts significantly reduced in SARS patients, but so was the proportion of NK cells expressing the receptor KIR2DL2/3 (CD158b) (National Research Project for SARS, Beijing Group 2004). Moreover, the number of NK and also KIR2DL2/3^+^ NK cells correlated with disease severity and anti-SARS coronavirus-specific antibodies. More recently, as previously observed for SARS-CoV-1 infection, severe COVID-19 cases exhibited lower counts of NK cells (Jiang et al., 2020; Qin et al., 2020; Sun et al., 2020; Wang et al., 2020b), especially COVID-19 patients admitted to ICU in comparison to no-ICU patients (Bordoni et al., 2020). Combined with the high infiltration of NK cells in the lung from mice infected with SARS-CoV-1 (Yao et al., 2020), these observations provide compelling evidence that NK cells and their receptors may be critical players for immune responses to coronaviruses.

The presence of KIR2DL2/3, whose expression on the NK cell surface was previously implicated with SARS (National Research Project for SARS, Beijing Group 2004), and of their HLA-C ligands were associated with hepatitis B (Gao et al., 2010; Di Bona et al., 2017; Auer et al., 2020). The extensive variation of the KIR genes and the associations with other viral diseases certainly make the KIR family a critical candidate for NK-cell-related COVID-19 studies. A recent meta-analysis (Leite et al., 2021) found no association between the presence/absence of KIR genes and COVID-19 case fatality rate. However, few studies addressed KIR in COVID-19 and much additional work is needed to ascertain if KIR haplotypes, genotypes, and alleles, as well as KIR/HLA compound genotypes are involved in the risk of SARS-CoV-2 infection and COVID-19 outcomes.

The NKG2A (NK group 2 member A) in another NK cell receptor that may be involved in the SARS-CoV-2 response. This receptor recognizes HLA-E as a ligand, suppressing NK cell cytokine secretion and cytotoxicity (Borrego et al., 1998; Braud et al., 1998; Lee et al., 1998). Moreover, NKG2A expression induces NK and CD8^+^ T cells to functional exhaustion in viral infections (Li et al., 2013; André et al., 2018). COVID-19 patients exhibited increased expression of NKG2A in comparison to controls, as well as characteristics of functional exhaustion of cytotoxic lymphocytes (Zheng et al., 2020). Like KIR, NKG2A is critical for the education of NK cytotoxicity in early developmental stages (Fauriat et al., 2010; Boudreau and Hsu, 2018; Zhang et al., 2019), and variation in KIR, NKG2A, and their HLA ligands could be responsible for differential immune responses against SARS-CoV-2.

## GWAS or Candidate Gene Approaches?

The position of HLA and KIR at the interface between hosts and pathogens, and the extensive list of associations between these loci and diseases, make them obvious candidates when looking for genetic variation associated with COVID-19. However, in the contemporary era of genomic studies, when the whole genome can be routinely queried to identify genetic variants contributing to a phenotype of interest, what could be the rationale for carrying out methods that focus specifically on a subset of loci (i.e., a candidate gene approach)?

The ability to analyze hundreds of thousands or millions of SNPs in microarray-based or NGS-based genome-wide association studies (GWAS) allows the discovery of multiple susceptibility loci in a single study. This exploratory approach is not based on hypotheses, thus permitting the identification of associations with variants that would not even be suspected to be involved in the disease. In fact, GWAS have already identified loci associated with phenotypes of SARS-CoV-2 infection (Ellinghaus et al., 2020; Pairo-Castineira et al., 2020). 

However, here we argue that GWAS as implemented leave out relevant variation at HLA, KIR, and other immune-related loci (also discussed in Kwok et al., 2020). These loci warrant the development of specific approaches to generate data for candidate gene studies, or that can be integrated into a GWAS, allowing to appropriately analyze their role in the host’s responses to infection by SARS-CoV-2 and the development of COVID-19.

Even though microarray-based methods can be cost-effective and provide genome-wide genotypic data, they can offer only a coarse map of associations. The SNP microarrays include a limited number of variants in comparison to the total existing variation, and a substantial portion of genetic variation is not captured. This may prove critical in analyses of HLA or KIR, where tag-SNPs in the microarray capture only a fraction of their variation. 

Another limitation that affects both SNP microarray-based GWAS and whole genome sequencing-based approaches is the existence of technical hurdles that preclude genotyping coverage for several immune-related regions. Many genes involved in immune responses are extraordinarily polymorphic and were originated by duplications or recombination events. For example, the uncommon structural variation of KIR and high homology among genes result in a lack of suitable reference alignments to include KIR specific SNPs in GWAS arrays. The extensive gene-content variation in KIR haplotypes is incompatible with pre-analysis quality control thresholds typically used in GWAS. Even the ImmunoChip, which was explicitly enriched for this region, only identifies non-coding variants of a single common KIR haplotype (Cortes and Brown, 2011). For example, associations between hepatitis B and C viral infections or diseases and KIR have been observed in candidate gene studies (Gao et al., 2010; De Re et al., 2015; Di Bona et al., 2017; Shan et al., 2018; Auer et al., 2020), but were missed in GWAS (Li et al., 2016; Vergara et al., 2019).

Consequently, the direct association of disease risk with KIR variation has only been detectable by targeted approaches. Other immune system genes, such as immunoglobulin genes, LILR, among others, are also poorly covered in GWAS for similar reasons (Horton et al., 2006; Calonga-Solís et al., 2019; Guselnikov and Taranin, 2019; Lefranc and Lefranc, 2020). HLA genes, on the other hand, may be imputed from SNP microarray data. However, as discussed below, this approach has limitations that may prevent the discovery of relevant associations. Although the MHC region is frequently observed in GWAS (Lenz et al., 2016), the associated HLA alleles or haplotypes are usually missed. Thus, for example, known associations of HLA and malaria were not found in GWAS (Kwok et al., 2020).

Taken together, these points suggest that generating data for KIR, HLA and other genes of the immune system using specific methods may be essential to obtain reliable data for these loci.

### Strategies for obtaining HLA and KIR data

The analysis of HLA and KIR loci and their effects on a complex phenotype such as COVID-19 can be carried out in three main frameworks. First, extracting SNP data from microarray-based genotyping methods (SNP microarrays). Secondly, when whole-genome sequencing (WGS) or whole-exome sequencing (WES) data are available, the sequence reads that align to the loci of interest can be selected and processed with appropriate bioinformatics tools to generate SNP and allele calls. Finally, it is possible to obtain HLA data from targeted sequencing through either amplicon or probe-based capture approaches, as many commercial HLA-genotyping kits also do. 

When using microarray-based genotyping data, it is possible to infer genotypes of different HLA and KIR genes using SNP data from surrounding regions, an approach known as imputation. The advantage of extracting HLA and KIR data from SNP microarrays is the possibility of analyzing these genes jointly with the genome-wide information, boosted with the relatively reduced costs of the SNP microarrays. Although powerful, HLA imputation is hampered in cases where there is a sparsity of informative markers, which varies among different SNP microarrays and platforms. Imputation methods have delivered HLA genotyping at two-field resolution (protein-level) with accuracy ranging from 89% to 98%, depending on the locus and the population (Zheng et al., 2014; Pappas et al., 2018; Geffard et al., 2020; Chen et al., 2021). Therefore, high-resolution genotypes may be missed, and the inaccuracies could eventually lead to false discoveries. For KIR, imputation methods from SNP microarrays are limited to the assessment of the presence and absence of specific genes (Vukcevic et al., 2015; Chen *et al.,* 2021) because the platforms usually include very few SNPs within the KIR region.

In addition, a major challenge to imputation is the informativeness of the reference panel (i.e., a large subset of samples with both the SNP microarray data and also HLA and KIR alleles genotyped by other methods) for the target sample. Despite efforts (Levin et al., 2014; Okada et al., 2015; Tian et al., 2016; Naslavsky et al., 2020), most reference panels are underrepresented for non-European and/or populations of mixed ancestry (Zheng et al., 2014; Neville et al., 2017), compromising the accuracy of HLA allele imputation for these groups (reviewed in Meyer and Nunes, 2017). For these reasons, there are worldwide initiatives to build better reference panels that include samples of under-represented populations, such as the Brazilian (Naslavsky *et al.,* 2020; Vince et al., 2020). 

In this context, WGS and WES data are more attractive - though costly - source of HLA and KIR data. However, the paralogy and extreme polymorphism make it challenging to obtain accurate HLA genotypes from WGS. Mapping short sequencing reads to reference genomes leads to mapping bias and loss of information (Brandt et al., 2015), potentially resulting in erroneous genotyping. Mapping bias is related to two different issues. First, reads carrying many nucleotide differences compared to the reference often fail to align, leading to an overestimation of reference alleles. Second, the cross-mappings between very similar genes reinforce the previous problem and result in the detection of false-positive variants. Bioinformatic approaches have been developed to overcome these difficulties for HLA genes: hla-mapper (Castelli et al., 2018) and MHC-PRG (Dilthey et al., 2015) improve mapping at the HLA region and accuracy of SNP calls; SNP2HLA (Jia et al., 2013), HLA*PRG (Dilthey *et al.,* 2016), HLA-VBSeq (Nariai et al., 2015), Kourami (Lee and Kingsford, 2018), HLAminer (Warren et al., 2012), and OptiType (Szolek et al., 2014) infer HLA alleles directly from short-read sequencing data. There are fewer methods available for KIR genes. KIR allele genotypes can be obtained from NGS data using the software PING (Norman et al., 2016; Marin et al., 2021), however the cost and computational efforts of analyzing WGS data is a limitation. 

Each method has pros and cons, depending on the data available, the number of samples, and the level of resolution needed. For instance, OptiType is highly accurate to predict two and three-field resolution alleles in a single sample basis. However, its database is outdated, and it fails to discriminate alleles differing only in introns and regulatory sequences. HLA-VBSeq also predicts HLA alleles for individual samples, but its accuracy is lower than other methods. Hla-mapper optimizes read alignment in HLA genes, allowing accurate detection of SNP-level genotypes in exons, introns, and regulatory sequences, at the cost of large sample sizes to obtain correct haplotypes. Another essential issue is the bias potentially introduced by hybridization-based capture panels not specifically designed for HLA and KIR genes (such as WES). The polymorphic nature of HLA and KIR may jeopardize the capturing, losing some exonic regions, or only capturing the segments that resemble the reference genome, thus leading to an overestimation of reference alleles. Although some of the methods presented above are compatible with WES, the use of exome data to determine HLA and KIR alleles should be carried out with caution, since the outcome might not represent the correct genotype distribution. Thus, accuracy of HLA and KIR allele calls may be lower for WES when compared to WGS unless specific panels designed for HLA and KIR genes are used.

Finally, targeted sequencing can provide the most precise high-quality data for HLA and KIR. However, this strategy lacks information that allows the analysis of HLA and KIR in combination with other regions of the genome. An interesting alternative is integrating targeted sequencing with a genome-wide approach. For example, Ellinghaus et al. (2020) used microarrays to perform a GWAS and applied targeted sequencing to genotype HLA for the association analysis with respiratory failure in COVID-19 patients.

None of these approaches for generating HLA and KIR data is universally preferable. The strategy to be used will depend on the research aims, available funding, synergy with other projects, and the intended speed of delivering results. Regardless of the approach, we emphasize that the complexity of HLA and KIR requires customized methods, or at least specific bioinformatic tools to process sequencing data.

### Analytical strategies for HLA and KIR in association studies

Our understanding of how HLA influences susceptibility and resistance to autoimmune and infectious diseases and how HLA and KIR genes have evolved, helps in planning the statistical analyses used in association studies. First, in addition to interrogating SNPs, it is possible to test individual HLA and KIR alleles or HLA supertypes (Ellinghaus et al., 2020), since these are more informative about the functional effects of the HLA molecules. Besides, it is feasible using HLA and KIR alleles as covariates to identify SNP associations that are independent of the HLA alleles or haplotypes (Kachuri et al., 2020).

Another strategy is to code individuals with respect to their heterozygosity over all classical HLA loci, the premise being that individuals that carry a larger number of distinct alleles are more likely to mount an effective response to viral epitopes. This last approach can be further refined by quantifying how much the alleles of an individual differ from each other at the amino-acid level, a measure which Arora et al. (2020) found to be correlated to HIV replication. Ellinghaus et al. (2020), in their association study of severe COVID-19 with respiratory failure, tested for both the multilocus heterozygosity and the amino acid divergence, but neither was significantly different between cases and controls in their study.

A final challenge for studies on host genetics of COVID-19 refers to the study design itself. While association studies of HLA or KIR and disease phenotypes are most frequently developed in a case-control format, it is by no means clear that this is appropriate for COVID-19. In many countries, including those most affected by the pandemic, the first set of GWAS were carried out at a time when less than 10% of the population had been infected. Thus, random population samples of unaffected individuals comprised a mixture of genetic backgrounds, ranging from possibly susceptible to resistant. This problem likely affected previous studies of SARS and MERS. However, it may be overcome for COVID-19 due to the substantially larger number of infected individuals, and the wide range of disease phenotypes. Accordingly, most studies have focused on comparing “extreme phenotypes”, a strategy that may increase the power to detect genetic effects. In this context, it is of utmost importance to carry on studies comparing the genetic background of individuals that have been exposed to the infection without developing the disease (e.g., individuals living in the same house as infected patients) and compare them to those who have contracted COVID-19.

## Host Genetics Beyond HLA and KIR

**Figure 4 -:**
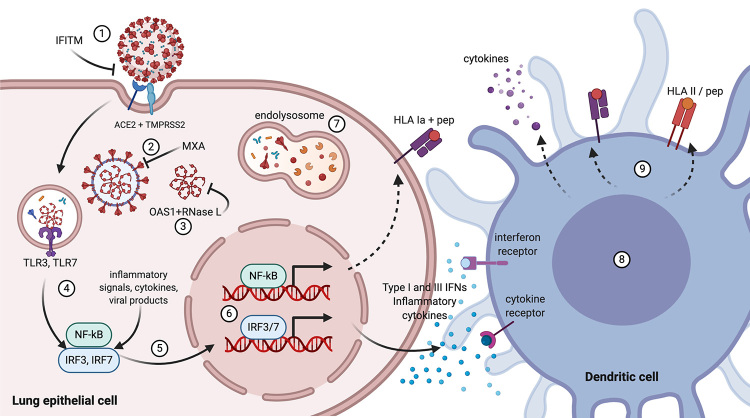
Some critical molecules involved in antiviral innate immune response, whose loss-of-function mutations and polymorphisms may result in severe COVID-19. SARS-CoV-2 entry into the cell is mediated by TMPRSS2 (transmembrane protease, serine 2) and the receptor ACE2 (angiotensin-converting enzyme 2). **(1)** The interferon-induced transmembrane proteins (**IFITM**) may inhibit the entry of viruses to the host cell cytoplasm. **(2)** The interferon-induced GTP-binding protein **MXA** may block endocytic traffic of incoming virus particles. **(3)** The 2’-5’-oligoadenylate synthase 1 (**OAS1**) binds ribonuclease L (**RNase L**) leading to its activation with subsequent degradation of the viral and cellular RNA, thus terminating viral replication. **(4)** Toll-like receptors 3 and 7 (**TLR3**, **TLR7**) recognize viral RNA leading to activation and **(5)** nuclear translocation of transcription factors NF-kappa-B (NF-kB) and IFN regulatory factors 3 and 7 (IFR3, IRF7). **(6) IFR3** and **IRF7** regulate the transcription of type I IFN genes (IFN-alpha and IFN-beta) and IFN-stimulated genes (ISG); NF-kB is a pleiotropic transcription factor crucial for regulation of numerous genes involved in immunity and other biological processes, such as apoptosis. **(7)** The lysosomal enzymes digest viral components in the phagolysosome. **(8)** Type I and III (IFN-lambda) interferons, NF-kB, and cytokines promote expression of numerous genes involved in innate and adaptive immune responses in different cells, including **(9)** up-regulation of **HLA** gene expression in antigen-presenting cells. Figure created with Biorender.

Apart from HLA and KIR, we will now focus on the genes and genetic systems involved in antiviral immune responses that may also be strong candidates for hypothesis-driven COVID-19 studies. Some of the proteins whose genetic variants may be involved in the infection by SARS-CoV-2 or COVID-19 are presented in Figure 4, in the context of an effective antiviral response. The examples we present do not intend to cover the full scope of the genetics of viral infections. Instead, they reveal a small portion of the complexity of the immunogenetics of infectious diseases.

### Cytokines and chemokines and their receptors

The cytokine release syndrome (CRS) or “cytokine storm” results from the over-production of soluble mediators of inflammation, which, in turn, sustain an uncontrolled systemic inflammatory response. The CRS characterizes a broad spectrum of non-infectious and infectious diseases, including SARS and MERS, and is common in patients with severe COVID-19 (Coperchini et al., 2020). CSR contributes to the disease’s pathophysiology, including hyper inflammation, thrombosis, hypotension, and pulmonary dysfunction in acute respiratory distress syndrome (ARDS) (Moore and June, 2020; Coperchini *et al.,* 2020). 

The levels of multiple cytokines and chemokines were significantly higher in SARS-CoV-2 infected patients and were associated with the severity of COVID-19 (Chi et al., 2020; Huang et al., 2020). Dysregulated activation of the mononuclear phagocyte compartment may contribute to the COVID-19-associated hyper inflammation (Merad and Martin, 2020). Activation of monocyte-derived macrophages by factors released from SARS-CoV-2 infected alveolar epithelial cells, activated T cells, and others, release massive amounts of IL-6 and other proinflammatory cytokines that initiate downstream signaling pathways. Also, the interaction of circulating activated monocytes and activated or damaged endothelial cells may trigger the extrinsic coagulation pathway, leading to intravascular blood clotting. This process can be amplified by the recruitment of neutrophils by the activated endothelial cells, which triggers the intrinsic / contact coagulation pathway (Merad and Martin, 2020).

Associations with variants in the genes encoding inflammatory cytokines, chemokines, and their receptors, have been reported for many diseases. They include anti-inflammatory cytokines or those that exert both pro- and anti-inflammatory effects, like TGF-b, IL-4, IL-10, and IL-27. Examples of viral diseases are the respiratory syncytial virus (RSV) infection (Gentile et al., 2003) and hepatitis B virus infections (Gusatti et al., 2016). No doubt, a comprehensive study of cytokine and chemokine genetic variation and expression levels will shed light on COVID-19 and its complications. 

A strong association between COVID-19 and respiratory failure with a genomic region at 3p21.31 was reported in a GWAS of Italian and Spanish patients with severe disease (Ellinghaus et al., 2020). The association signal revealed a cluster of several protein-coding and lncRNA genes, although the data could not reliably implicate a causal gene. However, three of the six protein-coding genes encode chemokine receptors, including the C-C motif chemokine receptor 9 (CCR9), the C-X-C motif chemokine receptor 6 (CXCR6), and the X-C motif chemokine receptor 1 (XCR1) (Ellinghaus *et al.,* 2020). Thereafter, results from two GWAS confirmed the association with this region at chromosome 3p21.31 (COVID-19 Host Genetics Initiative 2020) | GWAS meta-analyses round 5 2021; Shelton et al., 2021). This genomic region has a large effect on COVID-19 morbidity and mortality. The effects are similar to or larger than the ones of most established risk factors and are age-dependent, such that the risk is higher in younger individuals (Nakanishi et al., 2021). 

The associated genetic variants in 3p21.31 are all in strong linkage disequilibrium - LD (r^2^ > 0.98) and span almost 50 kb. This haplotype occurs in South Asia at a frequency of 30%, in Europe at 8%, and at lower frequencies in East Asia, but is absent in Africans. Therefore, the analysis of Africans could help to narrow-down the COVID-19 causal variant(s). Interestingly, the same extended haplotype was found in 50,000 to 120,000 old Neanderthal genomes, leading to the conclusion that it is inherited from Neanderthals (Zeberg and Pääbo, 2020). The unusually contrasting haplotype frequencies between South and East Asia indicate the effect of natural selection, possibly because of the exposure of these populations to different pathogens in the past. Concerning the SARS-CoV-2 pandemic, the chromosome 3 haplotype is now under negative selection with dramatic consequences (Zeberg and Pääbo, 2020).

### The complement system

The complement system comprises a network of dozens of soluble and cell membrane proteins that work in a coordinated manner towards the activation of three pathways - the classical, alternative, and lectin pathways (reviewed in Beltrame et al. (2015) and Reis et al. (2019)). These pathways are crucial in innate immunity and crosstalk with the adaptive response components, influencing disease outcomes. 

Both deficient and excessive activation of the complement molecules may be harmful to the host. Complement system deficiencies are frequently associated with increased susceptibility to infections and adverse manifestations in other diseases, especially in immunocompromised patients (reviewed in Beltrame et al., 2015; Reis et al., 2019). Conversely, abnormally increased levels of complement proteins, which are partially explained by genetic variation, may contribute to hyperinflammatory responses, observed also in severe COVID-19. Further, the complement system is also involved in other biological processes, including coagulation, which has been implicated in various COVID-19 complications (Bumiller-Bini et al., 2021). 

Some features of severe COVID-19 suggest that complement activation is possibly playing a critical role in the pathogenesis of this disease, particularly during exaggerated immune responses (Java et al., 2020). Genetic variants of several complement regulatory proteins and factors, including CD55(DAF), CFH, C3, C4BPA, and COLEC11, have been associated with adverse COVID-19 clinical outcomes (Ramlall et al., 2020). The comparison of postmortem lung biopsies of COVID-19 and H1N1 patients and control patients who died of causes not involving lung lesions showed higher expression of FCN3 (ficolin 3) in both diseases compared to the control group. The MBL2 (mannose-binding lectin) level was increased only in the COVID-19 group (Malaquias et al., 2020). Instead, low MBL2 serum levels and a variant of the gene *MBL2* were pointed out as risk factors for SARS-CoV-1 infection that causes the COVID-19-related disease SARS (Zhang et al., 2005).

### Toll-like receptors and genes involved in type I interferon regulated immunity

Cells detect pathogen-associated molecular patterns (PAMP) through pattern-recognition receptors (PRR), which allows semi-specific recognition of pathogenic microorganisms and viruses and influences innate and adaptive immune responses. The toll-like receptors (TLR) constitute one of the PRR classes (reviewed by Frazão et al., 2013). The surface receptors TLR1, TLR2, TLR4, TLR5, TLR6, and TLR10 are mainly responsible for detecting components from extracellular bacteria and fungi. However, these receptors also detect viral capsid proteins, including the SARS-CoV-2 S-protein (Choudhury and Mukherjee, 2020). Conversely, the intracellular TLR3, TLR7, TLR8, and TLR9 primarily recognize nucleic acids from viruses and bacteria. The TLRs upregulate anti-viral and pro-inflammatory mediators and, therefore, modify the infection’s outcome positively, limiting the viral load, or negatively, by triggering the exacerbated inflammation associated with the CRS.

In the Netherlands, whole-exome sequencing was performed for four young men hospitalized with severe COVID-19, all without a history of major chronic diseases. The two brother pairs were aged 32 and 29 (family 1, Dutch ancestry), 23 and 21 (family 2, African ancestry). One of the patients died. The study identified two different rare novel *TLR7* loss-of-function variants (van der Made et al., 2020). The analysis of men with severe COVID-19 aged less than 60 years found *TLR7* deleterious missense variants in 2.1% of the patients and in none of the asymptomatic participants (Fallerini *et al.,* 2021). In both studies, the *TLR7* variants were associated with impaired type I and II IFN responses. Recessive or incompletely dominant loss-of-function mutations of *TLR7* and other X-chromosomal genes such as *ACE2* (that encodes angiotensin-converting enzyme 2, the cellular receptor for SARS-CoV-2) and *NEMO* (encodes NF-kappa-B essential modulator, a regulatory protein involved in antiviral response) may be partly responsible for the higher risk of severe COVID-19 and higher death rates in men compared to women (Espinosa et al., 2020; Patil et al., 2020). Moreover, less detrimental common and rare variants of TLRs and other PRRs could contribute to the polygenic component of the COVID-19 susceptibility. 

Rare variants at 13 genes known to govern TLR3- and IRF7-dependent type I interferon (IFN) immunity to viruses were searched in 659 patients with severe COVID-19 pneumonia and 534 individuals with asymptomatic infection or mild disease. The study unveiled 24 loss-of-function variants underlying autosomal recessive or dominant deficiencies in 23 (3.5%) of critically ill patients, aged 17 to 77 (Zhang et al., 2020a). The 24 mutations were found in 8 of the 13 genes: *TLR3*, *UNC93B1* (protein unc-93 homolog B1 that regulates nucleotide-sensing TLR signaling), *TICAM1* (TIR domain-containing adapter molecule 1, a cytoplasmic viral sensor which participates in activation of transcription factors and induction of proinflammatory cytokines), *TBK1* (a multifunctional serine/threonine-protein kinase that plays an essential role in the TLR3- and IFN-dependent control of viral infections), *IRF3* and *IRF7* (IFN regulatory factors 3 and 7, key transcriptional regulators of type I IFN-dependent immune responses against DNA and RNA viruses), and *IFNAR1* and *IFNAR2* (IFN alpha/beta receptors 1 and 2, which associate to form the type I IFN receptor) (Zhang *et al.,* 2020a). Polymorphisms of *IFNAR2* and *TYK2* (involved in the initiation of type I IFN signaling) were also associated with critical COVID-19 in a recent GWAS of individuals of mostly European descent (Pairo-Castineira et al., 2020). Moreover, evidence in support of a causal link between low expression of *IFNAR2* and high expression of *TYK2* with life-threatening COVID-19 was reported (Pairo-Castineira *et al.,* 2020). Remarkably, life-threatening COVID-19 pneumonia can also result from auto-immune phenocopies of these inborn errors of type I IFN immunity. At least 10.2% (2.6% of women and 12.5% of men) of 987 critically ill COVID-19 patients had neutralizing IgG autoantibodies against type I IFNs; they were aged 25 to 87 years and 95 were men. None of the 663 subjects with asymptomatic SARS-CoV-2 infection or mild COVID-19 had these autoantibodies, which were present in only 0.0033% of 1,227 healthy individuals (Bastard et al., 2020).

### Proteins that antagonize viral entry into the host cell and replication

The 2’-5’oligoadenylate synthetase (OAS) protein family consists of the OAS1, OAS2, OAS3, and OAS-like (OASL) proteins. Type I and II IFNs induce synthesis of the OAS proteins that recognize exogenous nucleic acid to initiate antiviral pathways (Hovanessian and Justesen, 2007). The OAS1 is a tetrameric interferon-induced dsRNA-activated antiviral enzyme. It leads to dimerization and activation of ribonuclease L (RNase L), culminating in cellular and viral RNA degradation, thus inhibiting protein synthesis and viral replication. Alternatively, the antiviral effect can also be mediated via a pathway independent of RNase L (Uniprot | P00973, 2020). The paralogous genes *OAS1-3* are closely linked at the chromosomal position 12q24.13 and are involved in the same general function of inhibiting the early viral replication. The fourth member of the family, *OASL,* is located at 12q24.31 and has anti- and pro-viral dual functions, which depend on various mechanisms and the phase of the infection (Choi et al., 2015).

The SNP rs2660 G>A in *OAS1* was associated with SARS-CoV-1 infection in Han Chinese from Beijing. The allele rs2660*G conferred a dominant protective effect on SARS infection (He et al., 2006). Other viral infections are influenced by *OAS* gene variants or expression levels as well. *OAS1-OAS3-OAS2* haplotypes were associated with clinical outcomes of dengue virus infection in India (Alagarasu et al., 2013). The Zika virus (ZIKV) infection of A549 cells induces *OAS2* expression that inhibits ZIKV replication through enhanced IFNβ expression, which leads to the induction of the Jak/STAT signaling pathway (Liao et al., 2020). Association of critical COVID-19 with rs10735079 in the *OAS1-3* gene cluster was reported for a sample of mostly European ancestry in a recent GWAS (Pairo-Castineira et al., 2020) and increased levels of OAS1 decreases the susceptibility to COVID-19 and, in particular, severe COVID-19 (Hernández-Cordero et al., 2021). The splice-site variant rs10774671*G in the gene *OAS1* is associated with greater OAS1 expression and has strong alternative splicing quantitative trait loci (asQTL) effect on *OAS1* (Sams et al., 2016) that results in higher levels of the p46 isoform and reduced COVID-19 susceptibility and severity (Zhou et al., 2021). Interestingly*,* rs10774671 presents strong linkage disequilibrium (LD; r^2^ > 0.8) with other ~130 SNPs in non-Africans (including rs2669 and rs10735079 (cited above). In Africans, however, only weak LD is observed between rs10735079 and all other SNPs (r^2^ ≤ 0.5), according to LDlink (Machiela and Chanock, 2015). The reason for such distinct LD patterns among continental populations was explored and discussed in detail by Sams *et al.* (2016). The *OAS* genomic region exhibits an elevated frequency of Neandertal-derived alleles in non-African populations, despite the known purifying selection against Neandertal ancestry observed in humans (Sankararaman et al., 2014; Gallego Llorente et al., 2015). Therefore, the OAS Neandertal-introgressed haplotype was subjected to positive selection in human populations, possibly because it reintroduced the ancestral splice-site variant rs10774671*G (Sams *et al.,* 2016). This is a magnificent example of the relevance of studying population genetics and evolution of immune-related genes and highlights why differences between African and non-African populations must be considered in future studies of *OAS* variation and SARS-CoV-2 infection.

The members of the interferon-induced transmembrane (IFITM) family are antiviral cell-intrinsic restriction factors that inhibit the viral entry into the host cells by restricting the membrane fusion. The IFITM proteins are active against SARS-CoV-1, influenza A virus, Ebola virus, dengue virus, HIV-1, among others (Bailey et al., 2014), and therefore are candidates for genetic studies in SARS-CoV-2 infection. The three paralogous *IFITM1-3* genes are located at chromosome region 11p15.5. In a preliminary study of COVID-19, homozygosity for the *C* allele of rs12252 in the gene *IFITM3* was associated with more severe outcomes in an age-dependent manner (Zhang et al., 2020b). The splice-site variant rs12252 is also a 5’UTR and synonymous variant of *IFITM3,* as well as a long non-coding RNA (lncRNA) variant (Ensembl | rs12252, 2020; GTEx Portal | rs12252, 2020). Future studies should explore multiple tag SNPs along the gene-dense genomic region that hosts the three IFITM genes (Ensembl | IFITM genes region, 2020), besides the *NLRP6* (NLR family pyrin domain containing 6) gene that encodes the sensor component of the NLRP6 inflammasome and is involved in innate immunity and inflammation, and *IRF7*, already shown to be implicated in severe COVID-19 (see above).

The human myxovirus resistance protein MxA also plays an important role in the outcome of human viral infections. The intracellular MxA protein is induced by type I interferons and has broad activity against diverse RNA viruses and a few DNA viruses. *MX2* produces nuclear and cytoplasmic MxB forms and has potent activity against human immunodeficiency virus type 1 (HIV-1) and herpesviruses (Staeheli and Haller, 2018). The *MX1*, *MX2* and *TMPRSS2* (transmembrane protease serine 2, which is critical for SARS-CoV-2 host cell entry (Hoffmann et al., 2020), are closely linked at the chromosomal region 21q22.3 and linkage disequilibrium in that region is strong (r^2^ > 0.8) in non-African populations. The minor (less frequent) alleles of five SNPs correlated with high of *MX1* expression in blood and a reduced risk of developing severe COVID-19 in Europeans (Andolfo et al., 2021). Previously, polymorphisms in the *MX1* promoter associated with increased transcription *in vitro* were associated with decreased susceptibility to SARS in the Chinese Hong Kong population (Ching et al., 2010).

### The blood groups

The biological role of ABO (alternatively ABH) blood groups and their effects on infections, immunity, thrombosis, cardiovascular disease, and metabolism have been thoroughly reviewed (Nordgren and Svensson, 2019; Stowell and Stowell, 2019a,b). The synthesis of these histo-blood group antigens is mediated by fucosyl- and glycosyltransferases under the genetic control of *FUT2* (secretor), *FUT3* (Lewis), and *ABO*(*ABH*) genes. The variants of these genes are associated with susceptibility to infection and the severity of many human pathogens, including *Helicobacter pylori* and *Vibrio cholerae* (Stowell and Stowell, 2019a) and Norovirus (Nordgren and Svensson, 2019). The highly diverse Noroviruses (or Norwalk-like viruses) are the most common etiological agent of acute gastroenteritis worldwide, with the viral genotype GII.4 mostly implicated in human disease (Nordgren and Svensson, 2019). Disease susceptibility is dependent on the Norovirus genotype and is mediated by the presence or absence of the A, B, and/or H antigens on gut epithelial surfaces, the so-called secretor phenotype (Nordgren and Svensson, 2019). Non-secretor individuals, those having a nonsense mutation in *FUT2* that causes the expression of an inactive FUT2 enzyme, do not express these blood antigens in mucosal tissues and are also resistant to GII.4 and several other Norovirus genotypes. The *FUT2* protective allele is fully penetrant against infection as none of the non-secretor individuals develop Norovirus infection (Nordgren and Svensson, 2019).

A study of SARS demonstrated that patients from blood group O exhibited a lower risk of infection by SARS-CoV-1 when compared with non-O participants (Cheng et al., 2005). As to COVID-19, significantly decreased and increased risk was reported for individuals from blood groups O and A, respectively, in 2,173 hospitalized patients with a confirmed infection by SARS-CoV-2 in Wuhan and Shenzhen, China, compared to the frequencies in populations from the same regions (Zhao et al., 2020). Numerous studies in different populations followed. The consensus that emerged is that the association of COVID-19 with the ABO locus is highly significant and the risk of SARS-CoV-2 infection and possibly also COVID-19 severity is slightly lower for group O than for non-O groups (Ellinghaus et al., 2020; Liu et al., 2020; Pendu et al., 2021). 

Besides, associations between arterial and venous thromboembolic events with non-group O have been consistently observed in the literature. Interestingly, von Willebrand’s factor (vWF) and factor VIII levels are significantly higher in non-group O individuals, which might predispose them to pathologic clot formation (Stowell and Stowell, 2019b). In addition, there is evidence that ABO blood groups may affect vascular biology independently of, or in conjunction with, alterations in hemostasis (Stowell and Stowell, 2019b). For example, ABO blood group status may influence outcomes following acute vascular injury. Critically ill patients who presented a major trauma or severe sepsis were evaluated for ABO blood group status in addition to the development of acute respiratory distress syndrome (ARDS) or acute kidney injury (AKI). Among patients of European ancestry, but not African, blood group A individuals were more likely to develop ARDS or AKI (Stowell and Stowell, 2019b). In COVID-19, ARDS, AKI, and hemostasis alteration have also been reported (Wu et al., 2020, McIntosh, 2020). The analysis by a multi-omics approach suggested that the increased ABO protein level is a causal risk factor for COVID-19 susceptibility and severity (Hernández-Cordero et al., 2021). Clinical COVID-19 phenotypes, especially circulatory system complications, including thrombotic and coagulation-related phenotypes, were associated with genetically predicted increased ABO expression levels (Pathak et al., 2020).

### Genes involved in mucociliary clearance

Eight potential genomic regions (“super-variants”) associated with mortality by COVID-19 were identified in a GWAS with more than 18,600,000 SNPs (Hu et al., 2021). The white British patients’ sample included 1,096 SARS-CoV-2 infected cases, of which 292 were deaths and 804 were survivors. The disruption of DNAH7 (dynein heavy chain 7, axonemal) function may cause ciliary dysmotility and weakened mucociliary clearance capability, and CLUAP1 (clusterin associated protein 1) is required for ciliogenesis. This finding evidences the importance of respiratory cilia functioning properly in COVID-19 patients. Interestingly, *DNAH7* is the most downregulated gene after *in vitro* infection of human bronchial epithelial cells with SARS-CoV-2 (Nunnari et al., 2020). The protein WSB1 (WD repeat and SOCS box-containing protein 1) is involved in the innate immune response and antigen processing and presentation by HLA class I molecules and may enhance maturation of the IL-21 receptor, which is involved in NK and T cell functions. The other genomic regions contain variants related to thromboembolic disease, mitochondrial dysfunction, and cardiovascular disease (Hu *et al.,* 2021).

Proteins of the mucin family are components of the mucociliary clearance system, and therefore are crucial for innate defense. The mucin 5B, oligomeric mucus/gel-forming protein (MUC5B), is secreted in the lung, saliva, and cervix (Uniprot | Q9HC84, 2020). A polymorphism in the *MUC5B* gene is strongly associated with protein expression levels and susceptibility to some diseases. Patients with idiopathic pulmonary fibrosis (IPF) had significantly higher levels of MUC5B in the lung than controls, and the allele rs35705950*T was strongly associated with risk, especially in homozygosis (Seibold et al., 2011). This SNP is located upstream of the *MUC5B* transcription start site and is predicted to affect the binding affinity of different transcription factors and to be a splice-site variant of the long non-coding RNA *AC061979.1* gene that overlaps the *MUC5B* gene (Ensembl | *MUC5B* gene region, 2020). The allele rs35705950*T, a risk factor for IPF, was associated with protection against the development of severe COVID-19 in older adults (Fadista et al., 2021). The observed association with rs35705950 could be due to a protective effect of high mucin production in the airways, but the authors could not rule out a patient selection bias associated with the rs35705950 SNP (van Moorsel et al., 2020) Other mucin family members might also be candidates for studies of COVID-19 complications in the gut and airways.

## Consolidating Information

The importance of investigating genetic associations in diverse countries has recently become more evident (Hindorff et al., 2018), especially because the genetic basis of many complex phenotypes differs significantly among geographic regions (Martin et al., 2017). These differences may be explained by both geographic-related pathogen variability and variation in host genetics, which might influence the function of specific genes (reviewed in Domínguez-Andrés and Netea, 2019). Differential allele frequencies and linkage disequilibrium are the major factors responsible for discordant associations in different populations. For example, LD in regions 3p21.31 and 12q24.13 is strong and extended in Europeans, but absent in Africans and admixed Latin-American populations from the 1000G project (LDlink April 19, 2021). These regions harbor, respectively, a multigene cluster and the OAS gene cluster, both associated with severe COVID-19 in Europeans (see Section “Host genetics beyond HLA and KIR”). A clear example of discordant results due to contrasting allele frequencies between populations comes from HLA in endemic pemphigus foliaceus. Among other differences, the *HLA-DRB1* alleles associated with the highest risk in the Brazilian population of predominantly European ancestry are DRB1*01:02 and *04:04, but in Native Americans, highest risk is associated with DRB1*04:04 only, because DRB1*01:02 is not present (Petzl-Erler, 2020). Thus, the analysis of non-Europeans and admixed populations is urgently needed to identify the COVID-19 causal variants and to evaluate their impact on COVID-19 in worldwide populations. To this end, several collaborative initiatives and consortia have been launched and the first results are emerging (for example, Castelli et al. (2021); Castro et al. (2021); and Rede Genômica IPEC).

The extent of the admixture in Brazil poses challenges in study designing since ancestry heterogeneity is known to underlie spurious associations (Seldin et al., 2011; Thornton and Bermejo, 2014). The ancestry varies among Brazilian geographic regions, with a higher proportion of Native American contribution in the North, larger proportion of African in the Northeast, and a predominance of European background in the South and Southeast (reviewed by Souza et al. (2019). In addition, the ancestry of traditional communities such as *Ribeirinhos* and *Quilombolas* differs significantly from other populations within the same region (Kimura et al., 2013; Gontijo et al., 2018). 

Some reports suggest that ancestry may be associated with differential risk for COVID-19 (Sze et al., 2020; Andrasfay and Goldman, 2021; Shelton et al., 2021). However, it is challenging to disentangle the effects of ancestry from other confounding factors. For example, in countries such as the USA, UK, and Brazil, the non-European ancestry is associated with a lower socioeconomic status, which by its turn is also associated with higher incidence of some comorbidities (Franceschini et al., 2013; Musemwa and Gadegbeku, 2017) and with conditions that increase transmissibility and vulnerability. Among these conditions are overcrowded housing, occupation, access to healthcare, stress, unbalanced diets, use of public transport, and others (Hawkins, 2020; Sze *et al.,* 2020). Several studies indicate that COVID-19 is more severe and deadly in groups with lower socioeconomic status (Demenech et al., 2020; Figueiredo et al., 2020; Hawkins *et al.,* 2020; Clouston et al., 2021). The conditions associated with socioeconomic deprivation may interfere in the immunological response to COVID-19, especially in an exacerbated pro-inflammatory response and senescent phenotypes (Holuka et al., 2020).

Notwithstanding, other studies have found that non-European ancestries are associated with an increased risk of death by COVID-19, even after controlling for comorbidities and socioeconomic status (Harrison et al., 2020; Shelton et al., 2021). Contrasting frequencies of genetic variants that are implicated in differential susceptibility among populations is a plausible explanation for these results. However, it is still unclear if there are additional reasons for ancestry-related differential risk to COVID-19.

Investigating ancestry-associated risk may be even more challenging in studies of stratified admixed populations, in which it is necessary to adjust for population structure. This adjustment may eventually overcorrect and lead to a substantial loss in power to detect the effects of alleles that are geographically restricted and/or specific to certain ancestries (Martin et al., 2018). Consequently, studies of stratified populations require appropriate approaches that assume differential association according to genetic ancestry, as exemplified by admixture mapping and association mapping (Shriner, 2017; Skotte et al., 2019).

## Research Strategies and Community Engagement

In only a few months, an impressive abundance of HLA-focused COVID-19 studies have been initiated or are in advanced stages. While most of them focus on specific questions regarding the relationship between genetic variation and COVID-19 outcomes, there are a myriad of different approaches. Large biobanks, donor registries, and others are leveraging pre-existing data resources to analyze genetic variants involved in immune responses, genotyped either as targeted candidate genes or in genome-wide methods. While many HLA laboratories intend to perform high-resolution genotyping in COVID-19 cohorts, multiple strategies are being employed in collecting these cohorts. It is expected that several research groups will perform genotyping of cohorts from their local clinic or hospital. Others will examine their previously genotyped HLA or KIR data of transplant donors and recipients, recontacting those individuals or accessing their medical records to ascertain COVID-19 status. This strategy is also being employed by donor registries with pre-existing genotype data. Likewise, large biobanks with either high-density SNP genotyping data suitable for imputation of HLA, or WGS or WES data from which HLA genotypes can be extracted, are being examined in light of either medical records or patient self-reported COVID-19 disease, outcomes, or symptoms. 

An important endeavor to coordinate research activities is the *COVID-19 HLA & Immunogenetics Consortium* (CHIC). This effort unites the global community of HLA and immunogenetics experts and thought leaders to focus efforts, combine resources and share data to accelerate discovery in understanding the role of HLA and other immunogenetic loci in disease outcomes. This consortium currently numbers over 100 members, including HLA and immunogenetics-focused academic research scientists, HLA clinical laboratory directors, leaders of the major international immunogenetics scientific societies, Editors-in-Chief of immunogenetics-focused journals, and commercial vendors with HLA laboratory and bioinformatics products. A website describing these efforts and including information and resources for the research community can be found at hlacovid19.org. The intention of the web resources is to both centralize access to COVID-19-related HLA data through a dedicated database (HLA | COVID-19 Database portal) that includes HLA data-management and analysis tools, as well as to connect COVID-19 researchers and clinicians seeking HLA genotyping services with the immunogenetics laboratories that can provide them.

The database is being funded under an emergency United States National Institutes of Health (NIH) COVID-19 supplement to an existing grant. Under the parent grant, tools and services have been developed for the standardized analysis, collection, exchange and storage of immunogenomic data. Under the supplemental funding, efforts have begun to apply these tools and resources for HLA data management into this public database to promote data-sharing and accelerate discovery of the relationship between HLA variation and COVID-19. Because the complexity and extreme polymorphism of the HLA region make consolidation, equivalency, analysis, and biological interpretation of HLA data challenging, a centralized resource that aggregates data from disparate sources and platforms and provides well-curated bioinformatics and analytical tools will serve to accelerate discovery. This platform is intended to centralize access to COVID-19-related HLA data and HLA data-management and analysis tools. It will serve both as a knowledge base and technical resource for HLA and immunogenetics research on the COVID-19 pandemic. Many consortium members have already committed to depositing their data in the database.

Aside from the groups centered on HLA and KIR, numerous other international, national, and regional collaborative efforts have been established to discover the host genetic determinants of COVID-19 susceptibility, severity, and outcomes, and contribute to the knowledge of the biology of SARS-CoV-2 infection and disease. The most associated variants localize in genes involved in the innate and adaptive immune responses, as exemplified by the initial results of *The COVID-19 Host Genetics Initiative* commented above in the topic *Host genetics beyond HLA and KIR*.

## Concluding Remarks

The prime importance of the immune responses in susceptibility to viral infections urges deep investigation to understand the impact of host immunogenetics in COVID-19 pathogenesis.

The influence of HLA diversity on viral infections is well-documented. The involvement of HLA may occur along different paths, which includes the innate response through interaction with KIR and other less well-defined receptors of innate lymphoid cells (ILC). Additionally, HLA is directly involved in the adaptive response, playing a crucial role in antigen-presentation to CD4^+^ and CD8^+^ T-cells, with implications also for vaccine development. The great similarity between the paralogous genes and their astonishing polymorphism render the identification of genotypes at allele-level resolution a challenging endeavor for HLA and, especially, KIR, making targeted approaches and dedicated pipelines preferred over microarray-based or NGS-based GWAS.

Apart from HLA and their receptors, a vast collection of host molecules certainly also plays crucial roles in the immune responses to SARS-CoV-2 and COVID-19 pathogenesis. Included are viral restriction elements, toll-like receptors, cytokines and chemokines and their receptors, the complement system, among others. The genes of many (if not most) of these molecules present functional polymorphism. The GWAS as well as the screening of candidate genomic regions with a dense set of tag SNPs will identify genetic variants that modulate the host immune response to infection and COVID-19 manifestations and severity. Moreover, to uncover rare highly penetrant variants, i.e., genetic variants that follow a Mendelian or oligogenic quasi-Mendelian inheritance, segregation and linkage studies in families with unusual SARS-CoV-2 infection outcomes and the comparison of patients with contrasting extreme disease phenotypes are desirable.

For HLA and their receptors as well as for other genes that participate in immune responses, differences of allele and haplotype frequencies among populations, along with genetic variations among SARS-CoV-2 strains implicate the need for carefully studying populations of diverse geographic regions. To maximize the likelihood of discovering the disease-relevant genetic variants, patient samples covering the whole spectrum of SARS-CoV-2 infection outcomes, from asymptomatic to critical COVID-19 and characterized for disease complications, should be investigated in local and worldwide collaborations.

Integrative host immunogenetic studies will likely deliver ground-breaking insight into the pathogenesis of COVID-19, with considerable biological and clinical implications.
